# From Tooth Adhesion to Bioadhesion: Development of Bioabsorbable Putty-like Artificial Bone with Adhesive to Bone Based on the New Material “Phosphorylated Pullulan”

**DOI:** 10.3390/ma17153671

**Published:** 2024-07-25

**Authors:** Ko Nakanishi, Tsukasa Akasaka, Hiroshi Hayashi, Kumiko Yoshihara, Teppei Nakamura, Mariko Nakamura, Bart Van Meerbeek, Yasuhiro Yoshida

**Affiliations:** 1 Department of Biomaterials and Bioengineering, Faculty of Dental Medicine, Hokkaido University, Kita 13, Nishi 7, Kita-ku, Sapporo 060-8586, Hokkaido, Japan; nakanishi-ko@den.hokudai.ac.jp (K.N.); akasaka@den.hokudai.ac.jp (T.A.); 2 Section for Dental Innovation, Faculty of Dental Medicine, Hokkaido University, Kita 13, Nishi 7, Kita-ku, Sapporo 060-8586, Hokkaido, Japan; h.hayashi@den.hokudai.ac.jp; 3 Health and Medical Research Institute, National Institute of Advanced Industrial Science and Technology, 2217-14 Hayashi-Cho, Takamaysu 761-0395, Kagawa, Japan; kumiko.yoshihara@aist.go.jp; 4 Department of Applied Veterinary Science, Faculty of Veterinary Medicine, Hokkaido University, Kita 18, Nishi 9, Kita-ku, Sapporo 060-0818, Hokkaido, Japan; nakamurate@vetmed.hokudai.ac.jp; 5 School of Clinical Psychology, Kyushu University of Medical Science, 1714-1 Yoshinocho, Nobeoka 882-8508, Miyazaki, Japan; marin@phoenix.ac.jp; 6 KU Leuven, Department of Oral Health Sciences, BIOMAT & UZ Leuven, Dentistry, 3000 Leuven, Belgium; bart.vanmeerbeek@kuleuven.be

**Keywords:** phosphorylated pullulan, bioabsorbable polymers, artificial bone, bio-adhesion

## Abstract

Bioabsorbable materials have a wide range of applications, such as scaffolds for regenerative medicine and cell transplantation therapy and carriers for drug delivery systems. Therefore, although many researchers are conducting their research and development, few of them have been used in clinical practice. In addition, existing bioabsorbable materials cannot bind to the body’s tissues. If bioabsorbable materials with an adhesive ability to biological tissues can be made, they can ensure the mixture remains fixed to the affected area when mixed with artificial bone or other materials. In addition, if the filling material in the bone defect is soft and uncured, resorption is rapid, which is advantageous for bone regeneration. In this paper, the development and process of a new bioabsorbable material “Phosphorylated pullulan” and its capability as a bone replacement material were demonstrated. Phosphorylated pullulan, which was developed based on the tooth adhesion theory, is the only bioabsorbable material able to adhere to bone and teeth. The phosphorylated pullulan and β-TCP mixture is a non-hardening putty. It is useful as a new resorbable bone replacement material with an adhesive ability for bone defects around implants.

## 1. Introduction

Autogenous bone has the highest bone regenerative capacity among bone replacement materials for treating bone defects [1]. Autogenous bone contains many components important for bone formation, such as calcium phosphate, collagen, bone morphogenetic proteins (BMPs), and cells associated with bone formation. However, the amount of autogenous bone that can be harvested is limited, so artificial bone made of calcium phosphate is widely used as an alternative material because it has inorganic components and a crystal structure similar to natural bone [2,3]. However, the ability of this material to regenerate bone is inferior to that of autogenous bone. Also, in dental clinical practice, granule-type artificial bone with a particle diameter of several hundred µm is used. The resorption of artificial bone to replace natural bone occurs more rapidly with fine particles than with larger granules, resulting in the faster healing of bone defects. However, fine particles induce inflammation [4]. In addition, when they enter blood vessels, they may move into the bloodstream and accumulate in the body’s organs. Thus, granular artificial bones currently on the market have a particle diameter of several hundred µm to several mm because they have been developed based on products that have obtained regulatory approval.

Nonetheless, new medical technology cannot advance by merely following predecessors. Different approaches from existing products are required to develop materials with novel functions. For this purpose, a collaboration with different fields is essential. Therefore, we used a strategy to develop bioabsorbable polymers that adhere to bone based on basic research on tooth adhesion. Here, we describe a novel bone replacement material with bone-adhesive properties and enhanced resorbability in the body.

## 2. Current Artificial Bone

There are various types of artificial bones made of calcium phosphate, such as granules, blocks, and hardened pastes. Granular artificial bones are the most common in dental clinical practice. Block-type artificial bone is intended to maintain its shape but could be slow to be absorbed in the body. Hardened pastes should be slightly resorbed because areas in contact with blood are insufficiently hardened, but the hardened body is basically nonabsorbable. Initially, non-absorbable hydroxyapatite was used for granular artificial bone. However, as the demand for resorption and bone replacement increased for artificial bone, β-tricalcium·phosphate (β-TCP) granules were introduced into clinical practice as an absorbable artificial bone. Subsequently, artificial bones that consisted mainly of apatite carbonate—the same inorganic component of natural bone [5]—and octacalcium phosphate (OCP), a precursor of apatite carbonate [6,7], were developed.

### 2.1. Hydroxyapatite

Hydroxyapatite (Ca_10_(PO_4_)_6_(OH)) has a composition similar to that of bone. Therefore, it is highly biocompatible and can adhere directly to bone tissue during bone formation when used as a bone replacement material [8]. Nevertheless, simply synthesized hydroxyapatite does not contain trace elements such as Na^+^ and Mg^2+^, which are found in real bone. Over the past few decades, many researchers have demonstrated that adding various ionic substituents to synthetic hydroxyapatite can produce a mineral composition similar to that of natural bone tissue [9,10,11,12]. Furthermore, synthetic hydroxyapatite cannot reproduce the porosity that distinguishes it from natural bone. Synthesized hydroxyapatite is characterized by very slow or no absorption, due to a high Ca/P rate and crystallinity [13]. A limitation of using hydroxyapatite as a bone replacement material is its low mechanical strength and fragility. Nanotechnology is an important solution to this fragility. Reducing the size of the crystalline grain of hydroxyapatite to a smaller or nano-sized one can decrease internal pores and defects. Moreover, material plasticity can be improved by increasing the number of grain boundaries [14]. Zhao et al. successfully improved carbon nanofiber-reinforced hydroxyapatite by reducing the size of the hydroxyapatite grain using spark plasma sintering [15]. Zeng et al. also reduced the grain size of hydroxyapatite and improved its mechanical properties by creating hydroxyapatite using a two-step sintering process in the digital light processing of 3D printing technology [16].

### 2.2. β-Tricalcium Phosphate (β-TCP)

β-TCP (β-Ca_3_(PO_4_)_2_) has been widely used as a bone replacement material for more than 30 years [17,18]. It has good osteoconductivity due to its porosity, which promotes vascular fiber growth and osteogenic cell adhesion [13,19]. β-TCP also has low immunogenicity in the body and high biosafety [13]. As described earlier, it possesses excellent properties as a bone replacement material. In their study of dog alveolar bone, Nakajima et al. found that the bone regenerative potential of β-TCP was comparable with that of freeze-dried heterologous bone, autogenous bone, demineralized freeze-dried bone, and inorganic bovine bone [20]. Galois et al. considered the use of β-TCP an optimal choice for moderate bone defects [18]. β-TCP is considered to be absorbed by osteoclasts, and its Ca/P rate is lower than that of hydroxyapatite, resulting in faster degradation and absorption in the body than hydroxyapatite [21]. However, its absorption rate is said to be unpredictable [22]. Furthermore, the interconnected porous structures of β-TCP can improve vascularization; however, the mechanical strength is reduced [23], thereby limiting applications and sites of use [17]. Recent studies have attempted to improve the mechanical strength of β-TCP using liquid phase sintering to increase the density of the material [24,25].

### 2.3. Carbonate Apatite

Carbonate apatite is the inorganic composition of bone, and the material was developed based on the idea that a material with a composition similar to that of bone would be best suited for use as a bone replacement material. Nevertheless, it was initially difficult to develop carbonate apatite because it pyrolyzes and therefore cannot be sintered. When calcium carbonate blocks are immersed in a phosphate solution, carbonate apatite precipitates on the block surface. This reaction progresses over time, transforming the entire block into carbonate apatite [5]. Using this technique, carbonate apatite can be produced in a clinically usable form. Carbonate apatite dissolves in the weak acid produced in the osteoclast Howship’s lacuna, similar to bone, and is absorbed faster than hydroxyapatite [26]. Clinical trials have shown that carbonate apatite is an effective bone substitute in human sinus lifts [27]. Carbonate apatite also possesses an excellent bone-conducting ability. It has been reported that carbonate apatite has better bone formation around it in beagle dogs than hydroxyapatite and possesses a superior osteoconductive ability [28]. This superior osteoconductivity is due to intercellular signaling from osteoclasts. In other words, osteoclasts that absorb carbonate apatite release liquid factors known as clastokines that activate osteoblasts [29]. Hydroxyapatite is not absorbed or is absorbed slowly, causing this phenomenon to rarely occur. Moreover, osteoblasts are activated in carbonate apatite through information transfer between the material and the cells. When bone marrow cells are seeded on the surface of carbonate apatite and analyzed for differentiation markers to osteoblasts, they show higher values than those on the hydroxyapatite surface [30]. In recent years, carbonate apatite has been verified to be more porous in order to improve its osteogenic potential [31].

### 2.4. Octacalcium Phosphate (OCP)

OCP (Ca_8_H_2_(PO_4_)6-5H_2_O) was suggested as a precursor for hydroxyapatite formation from an aqueous solution [6] and for apatite crystal formation during mineralization [7]. OCP is composed of a repetitive apatite layer, similar to that in Ca-deficient hydroxyapatite, and a hydrated layer containing large amounts of water molecules, such as dicalcium phosphate dihydrate (DCPD) [7]. It was anticipated that OCP could be used as a bone replacement material because of its potential to function as a precursor phase, as observed in the formation of OCP before hydroxyapatite deposition in bone collagen matrices in natural bone [32]. When implanted in the mouse calvaria, OCP demonstrated earlier bone formation than hydroxyapatite and Ca-deficient hydroxyapatite. Furthermore, bone apatite crystals are bonded by OCP crystals during bone formation [33]. The degradation of OCP might be primarily caused by the phagocytosis of osteoclasts because OCP induces osteoclast formation in an in vitro coculture system of osteoblasts and osteoclast precursor cells [34]. The degradation rate of OCP is faster than that of hydroxyapatite and tricalcium phosphate [35]. Based on these characteristics of OCP, a human clinical trial was conducted using a mixture of OCP and collagen, which demonstrated effective results in cases of sinus floor elevation in the 1st and 2nd stages, socket preservation, cyst, and alveolar cleft procedures [36]. Various forms of OCP, including granules [37] and blocks [38], and various additives, such as biodegradable polymers [36,39,40] and ions [41,42], are now considered to improve the effectiveness of OCP.

## 3. Problems and Solutions of Existing Artificial Bone

**Figure 1:**
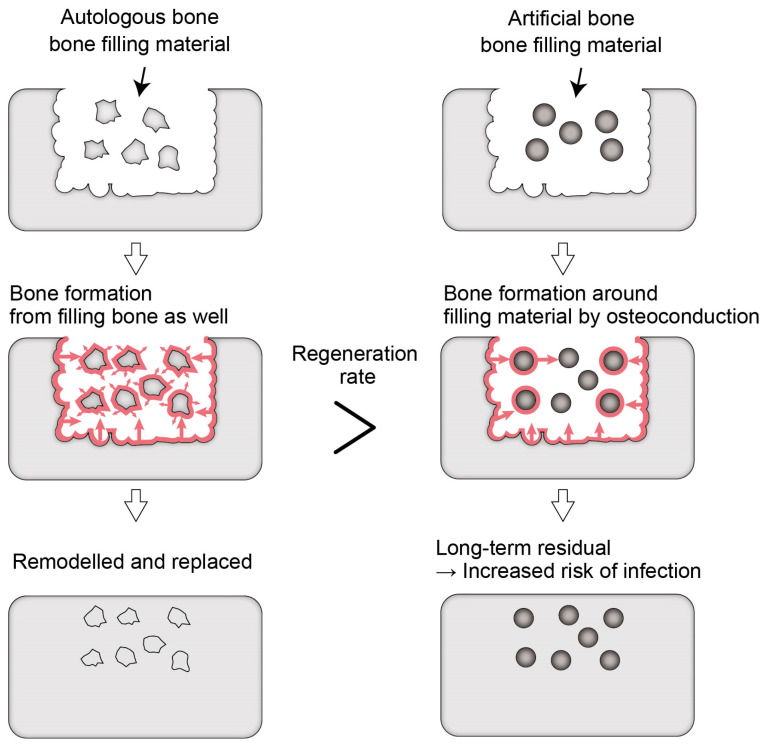
Differences between autogenous bone and artificial bone in terms of bone formation and resorption patterns. Red arrows show the direction of bone regeneration.

These artificial bones made of calcium phosphate are osteoconductive [43], as mentioned above. Bone regeneration occurs around granules with a size of several µm before they are resorbed, and the artificial bone remains in the bone. Therefore, it is suggested that even β-TCP, carbonate apatite, and OCP also take a long time to be completely absorbed and replaced by natural bone in the human body. The larger the particle size of the resorbable artificial bone, the longer it takes for its resorption and replacement by natural bone in the human body. This may help reduce bone resorption but is a major problem for controlling infection because the implanted material that remains in the body increases the risk of infection (Figure 1). For example—as is evident in catheter infections—it is challenging to control infection when an implanted artificial material is exposed to the outside of the body and becomes infected. The same happens to artificial bones. When the infection on artificial bones cannot be controlled, they must be removed by curettage.

Furthermore, granular artificial bones are difficult to handle. Mixing with saline or blood improves handling but only slightly. The size of bone defects in dentistry is smaller than in orthopedics, and the particle size of the granular artificial bone used in dentistry is also smaller. Besides the oral cavity being a site of easy infection, osteogenesis of the alveolar ridge requires placing a granular artificial bone to fit the defect, which increases the procedure’s difficulty. In addition, a smaller granular artificial bone results in greater risks of dispersal, migration, and leakage through the incision.

## 4. Necessity of Bioabsorbable Polymers that Adhere to Calcium Phosphate

A bioabsorbable material that can adhere to bone and artificial bone components is desirable because it would allow the artificial bone to be easily placed in a bone defect by mixing the artificial bone with it and making it patty-like. In addition to the improved handling due to enhanced formability and adhesion to the bone surface, the stronger sealing of the natural bone–artificial bone interface is expected to improve the treatment outcome. Furthermore, if the material that adheres to calcium phosphate can maintain its gel state in the body, and if fine powdered artificial bone can be used, it leads to the development of new artificial bone able to be quickly absorbed and replaced with natural bone. The rapid resorption of bone replacement materials and their substitution with natural bone is ideal for reducing the risk of infection.

## 5. Problems with Existing Bioabsorbable Polymers

For the above reasons, the development of absorbable polymers that can adhere to calcium phosphate, which is an inorganic component of bone and is used as artificial bone, has been long awaited. If such materials are available, innovative materials that contribute to implant therapy, such as putty-like artificial bones with short-term resorption and replacement by mixing a bioabsorbable polymer and calcium phosphate fine powder, are expected to be developed. However, materials that can be absorbed and replaced with tissue in the body are challenging to put to practical use. Collagen, hyaluronic acid, polyglycolic acid, and polylactic acid are still widely used as absorbable polymers that can be implanted in the body, but none of them can adhere to bone or tooth.

## 6. Development of Non-Animal-Derived Bioabsorbable Polymers

Phosphorylated pullulan was developed to solve problems with the above existing artificial bones. The raw material, pullulan, is a natural polysaccharide produced by fermentation with black yeast; it has water-soluble properties among polysaccharides and can be processed into powder and film. Pullulan has been used in the food industry for many years and was introduced as a non-gelatin capsule material in pharmaceuticals because its firm had excellent gas barrier properties when bovine spongiform encephalopathy became a major problem in Japan [44].

We hypothesized that introducing numerous phosphate groups into pullulan would yield a bioabsorbable material that adheres to bone (Table 1).

## 7. Design Concept of Phosphorylated Pullulan

Mechanical mating between the adherend and the material and chemical bonding at the interface are important factors when considering adhesive materials. Mechanical mating is considered to affect the adhesive strength in tooth bonding, whereas chemical bonding contributes to bond durability [45]. It is also a concept agreed upon by all those involved in adhesive bonding that adhesive materials need to have adequate wettability to achieve a high adhesive strength. Pullulan is easily soluble in water, and the viscosity of a pullulan aqueous solution is very low among polysaccharides. Using pullulan as a backbone reduces viscosity, compared to other polysaccharides, leading to the creation of a bioabsorbable polymer with adequate wettability.

Phosphorylated pullulan was created based on the tooth adhesion theory, using pullulan as a backbone. In the dental field, the progress of tooth adhesion is particularly remarkable. It is well known that adhesive techniques and materials are applied to most current dental treatments, such as aesthetic restorations, including resin fillings, posts and cores, crown restorations, orthodontics, fixing mobile teeth, and bonding fractured teeth. Many dental adhesives have been developed and applied in clinical practice with repeated improvements and development. Nowadays, two types of adhesives (glass ionomer cement and resin cement) are commonly used in dental treatment.

Glass ionomer cement is cured by an acid-base reaction between polycarboxylic acid and the cations leached from aluminosilicate glass and can chemically bond well to untreated enamel and dentin. Thus, glass ionomer cements have the ability to chemically bond to the tooth [46]. Compared to resin-based tooth-bonding systems, glass ionomer cement can be used even in areas with insufficient moisture protection, which is thought to be largely due to the characteristics of polycarboxylic acid (the main component). Polycarboxylic acid has many carboxy groups that chemically bond to teeth. In addition, many of these carboxyl groups are considered to make glass ionomer cements somewhat hydrophilic and not as hydrophobic as resin-based tooth-bonding systems.

**Figure 2:**
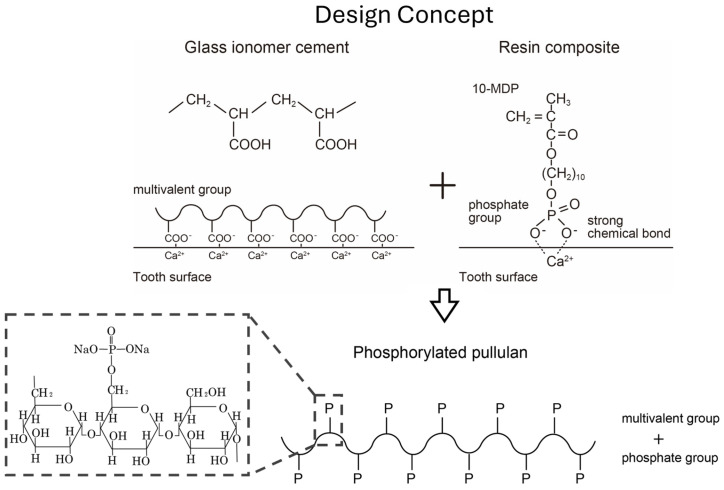
Design concept.

Furthermore, we previously compared three adhesive monomers: 4-methacryloxyethyl trimellitic acid (4-MET), 2-methacryloxyethyl phenyl hydrogen phosphate (phenyl-P), and 10-methacryloyloxydecyl dihydrogen phosphate (10-MDP) and found that 10-MDP had the best chemical bonding ability [47]. Since this report, the effectiveness of the divalent phosphate group has become clear, and 10-MDP has been used in various tooth-bonding systems. These results of basic research on tooth adhesion [48,49,50,51,52] allowed for the development of phosphorylated pullulan, based on the idea of “introducing a large number of phosphate groups into a polysaccharide with high water solubility” (Figure 2).

## 8. Efficacy of Phosphorylated Pullulan as a Bone Replacement Material

Phosphorylated pullulan has been applied in various ways, due to its characteristics, such as a coating agent for implants [53,54,55,56] and as a direct pulp-capping material [57,58,59]. Takahata et al. [60] and Morimoto et al. [61] reported the efficacy of phosphorylated pullulan as a bone replacement material (Table 2).

Takahata et al. [60] implanted a mixture of phosphorylated pullulan and β-TCP in the medullary cavity of a mouse femur, a rabbit ulnar, and a porcine vertebral body bone and evaluated bone formation. A histological evaluation of the medullary cavity of a mouse femur revealed that only phosphorylated pullulan remained 2 weeks after implantation. Five weeks after implantation, bone marrow replacement was observed in the group implanted with only phosphorylated pullulan, and bone formation was observed in the group implanted with a mixture of pullulan phosphate and β-TCP. After 8 weeks, additional new bone was observed in the group implanted with a mixture of phosphorylated pullulan and β-TCP, but no bone formation was observed in the group implanted with only phosphorylated pullulan. The effects of Biopex-R and a mixture of phosphorylated pullulan and β-TCP in the rabbit ulnar were compared. Eight weeks after implantation, a micro-CT showed that Biopex-R separated from the existing bone in the group implanted with only Biopex-R, but the bone defect was completely amended in the group implanted with a mixture of phosphorylated pullulan and β-TCP. Histologically, the type of this bone formation is classified as endochondral ossification. On the other hand, the formation of new bone in the porcine vertebral body bone in the group with no treatment for the defect and the group implanted with Biopex-R was not confirmed by micro-CT, even after 8 weeks. By contrast, a mixture of phosphorylated pullulan and β-TCP implanted in the bone defect of the porcine vertebral body bone resulted in new bone formation at 8 weeks. Histologically, only fibrotic changes were observed in the untreated group, even at 8 weeks. However, in the group implanted with a mixture of phosphorylated pullulan and β-TCP, the new bone began to form at 4 weeks, and trabecular bone formation began at 8 weeks. Based on this, Takahata et al. concluded that phosphorylated pullulan alone cannot induce bone formation, but when used together with β-TCP, it promotes bone remodeling.

**Figure 3:**
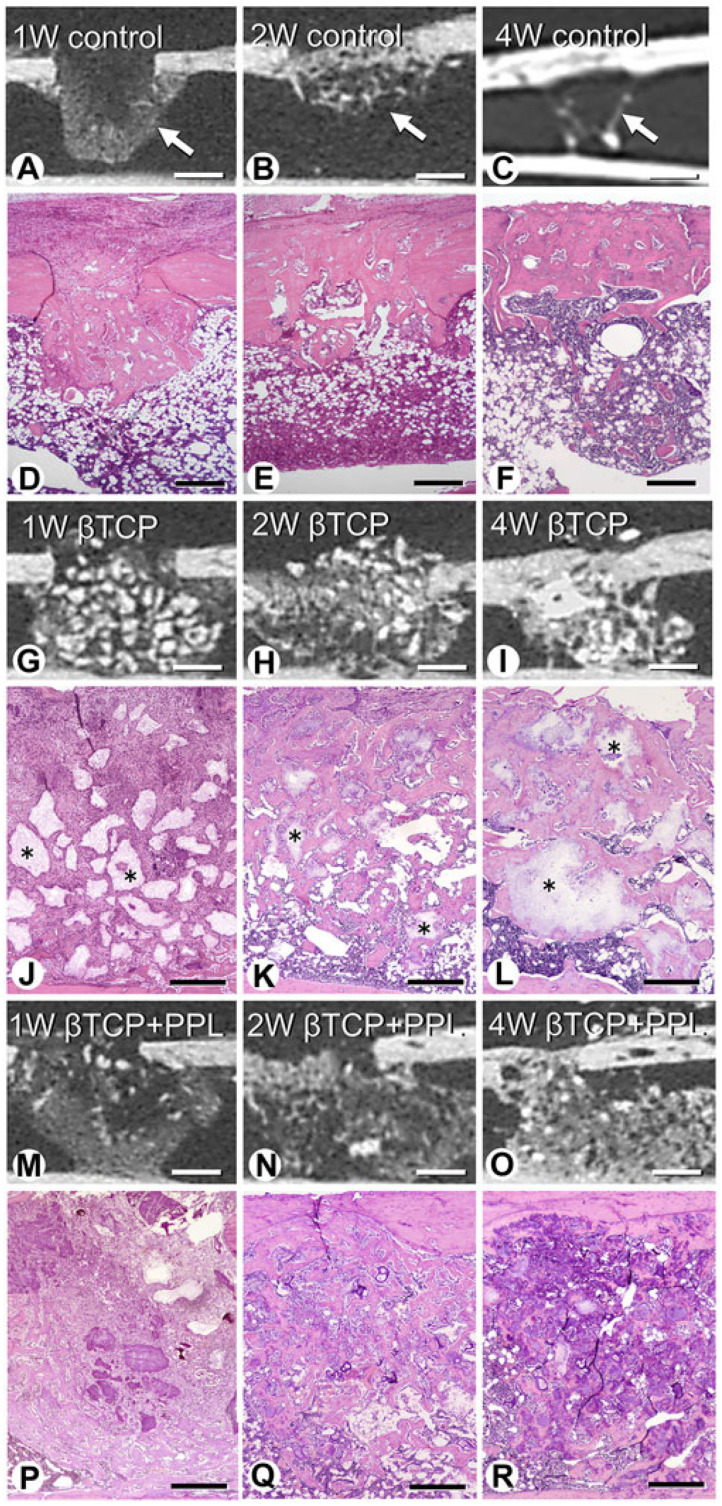
Micro-CT images and histological images after the implantation of β-TCP or β-TCP and phosphorylated pullulan in rats with a tibia bone defect. (**A**–**C**): Micro-CT images of the control group. (**D**–**F**): Histological images of the control group. (**G**–**I**): Micro-CT images of the β-TCP implantation group. (**J**–**L**): Histological images of the β-TCP implantation group. (**M**–**O**): Micro-CT images of the group implanted with a mixture of phosphorylated pullulan and β-TCP. (**P**–**R**): Histological images of the group implanted with a mixture of phosphorylated pullulan and β-TCP. *: β-TCP. Bar, (**A**–**C**,**G**–**I**,**M**–**O**): 1 mm, (**D**–**F**): 400 μm, (**J**–**L**,**P**–**R**): 300 μm. (Morimoto et al. Frontiers in Bioengineering and Biotechnology, 2023, 11 [61]).

**Figure 4:**
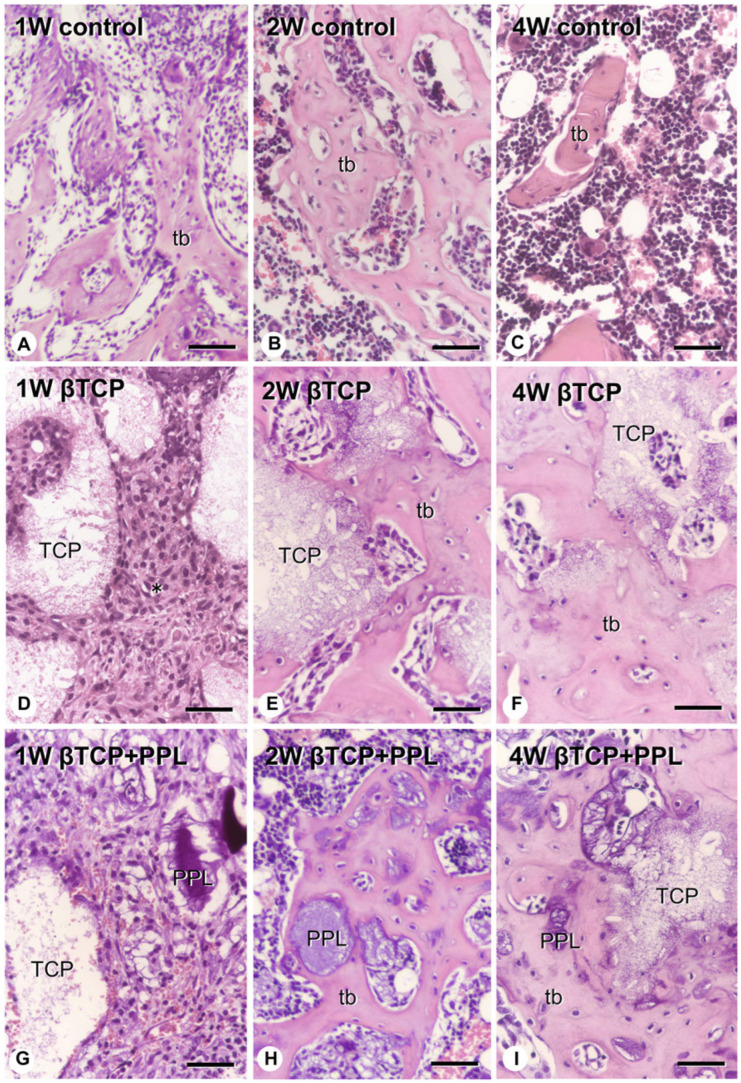
High-magnification histological images after the implantation of β-TCP or β-TCP and phosphorylated pullulan in rats with a tibia bone defect. (**A**–**C**): Control group. (**D**–**F**): β-TCP implantation group. (**G**–**I**): Group implanted with a mixture of phosphorylated pullulan and β-TCP. tb: trabecular bone, TCP: β-TCP, PPL: phosphorylated pullulan. Bar: 30 μm (Morimoto et al. Frontiers in Bioengineering and Biotechnology, 2023, 11 [61]).

Morimoto et al. [61] implanted a mixture of phosphorylated pullulan and β-TCP in rats with a tibia bone defect, analyzed the bone regeneration process histologically and microstructurally, and reported that phosphorylated pullulan may have osteoconductive and calcification retention properties. These authors created a cylindrical bone defect with a diameter of 2.0 mm in the tibia of 10-week-old rats, and two materials, namely β-TCP (mean granule diameter = 100–250 μm) and a mixture of pullulan phosphate and β-TCP (4:6, *w*/*w*), were implanted in the animals and compared. A group with the bone defect but without an implant was used as the control. In the control group, a large amount of new bone was observed in the bone defect after 1 week, but these new bones decreased 4 weeks after implantation. By contrast, in the β-TCP implantation group, β-TCP granules were surrounded by numerous fibroblast-like cells 1 week after implantation; then, new bone was formed at 2 to 4 weeks, using β-TCP as a scaffold. This new bone remained in the bone defect 4 weeks after implantation. In the group implanted with a mixture of phosphorylated pullulan and β-TCP, numerous fibroblast-like cells were observed around phosphorylated pullulan and β-TCP 1 week after implantation, and new bone was observed on the phosphorylated pullulan and β-TCP granules after 2 weeks (Figure 3 and Figure 4).

**Figure 5:**
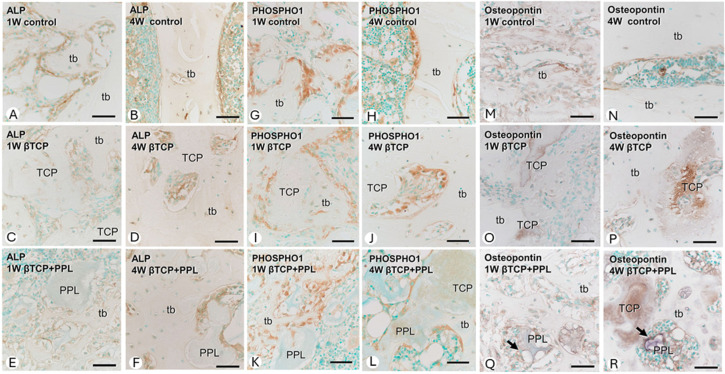
Immunohistochemical staining images of ALP, PHOSPHO1, and osteopontin. (**A**–**F**): ALP, (**G**–**L**): PHOSPHO1, (**M**–**R**): osteopontin. tb: trabecular bone, TCP: β-TCP, PPL: phosphorylated pullulan, Arrow: immunohistochemical reaction of PPL surface, Bar: 30 μm (Morimoto et al. Frontiers in Bioengineering and Biotechnology, 2023, 11, partially modified [61]).

Osteoblast lineage cells and bone formation at the bone regeneration site were evaluated by immunohistochemistry for alkaline phosphatase (ALP) (Figure 5A–F), PHOSPHO1 (Figure 5G–L), and osteopontin (Figure 5M–R). In the control group (without implantation), numerous ALP-positive osteoblast lineage cells (osteoblasts and preosteoblasts) were present on the surface of new bone during all postoperative periods. Moreover, PHOSPHO1-positive reactions—expressed on osteoblasts responsible for matrix vesicular calcification—were also observed on the bone surface. In the β-TCP implantation group, ALP-positive osteoblast lineage cells and PHOSPHO1-positive osteoblasts were found on the surface of new bone and on β-TCP granules. Thereafter, ALP-positive osteoblast lineage cells were more widespread on the surface of new bone than PHOSPHO1-positive osteoblasts. In the group implanted with a mixture of phosphorylated pullulan and β-TCP, ALP-positive osteoblast lineage cells and PHOSPHO1-positive osteoblasts were located not only on the surfaces of β-TCP and new bone but also on phosphorylated pullulan. Interestingly, osteopontin, a bone matrix protein able to bind to crystalline calcium, was found on the surfaces of β-TCP and phosphorylated pullulan, suggesting that phosphorylated pullulan may function as a scaffold material for osteoblast fixation. In addition, an analysis using strain-derived osteoblasts (MC3T3-E1 cells) suggested that phosphorylated pullulan did not have a direct effect on osteoblast differentiation or function.

On the other hand, bone resorption was evaluated by osteoclast distribution using tartrate-resistant acid phosphatase (TRAP) staining. Osteoclasts were most abundant 2 weeks after implantation in all groups and then decreased. In the control group, TRAP-positive osteoclasts were located on the surface of the new bone, whereas in the β-TCP implantation group, they were located not only on the new bone but also on β-TCP. In the group implanted with a mixture of phosphorylated pullulan and β-TCP, the TRAP-positive osteoclasts were mainly located on the surface of the new bone, but some osteoclasts were also localized on the surface of phosphorylated pullulan.

**Figure 6:**
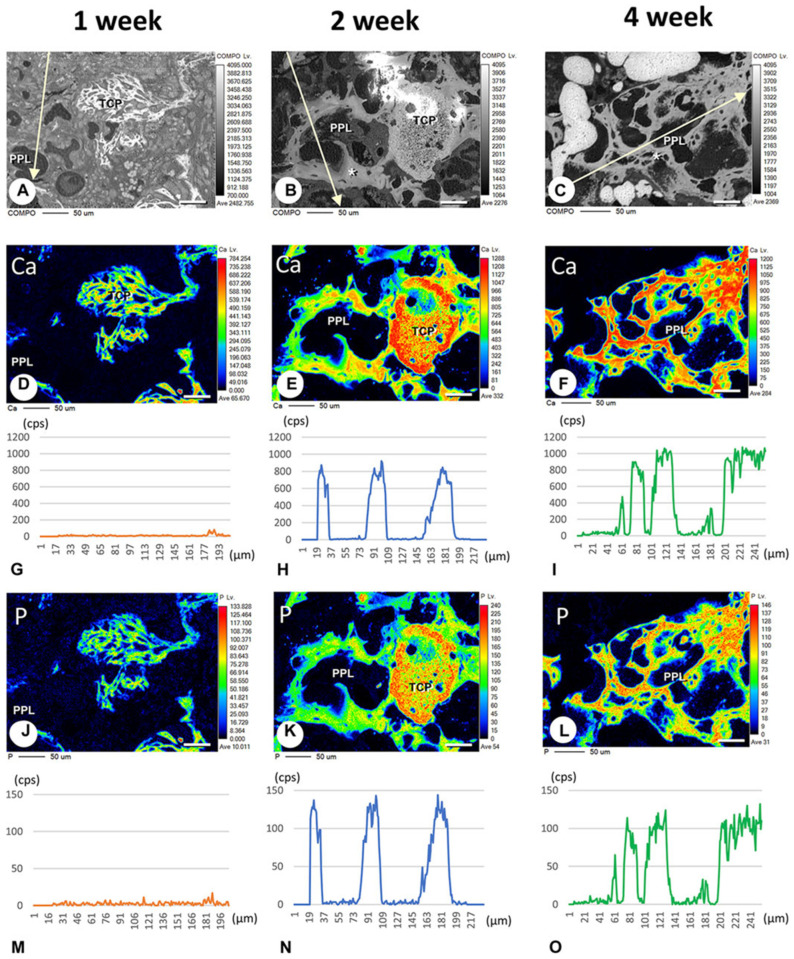
Results of the electron probe micro-analyzer measurement after the implantation of β-TCP and phosphorylated pullulan in rats with a tibia bone defect. The composition (COMPO) images of the bone defect region at 1, 2, and 4 weeks after the implantation of β-TCP and phosphorylated pullulan are illustrated. (**A**–**C**) The electron probe micro-analyzes images of Ca (**D**–**F**) and P (**J**–**L**) are shown in (**D**–**F**) and (**J**–**L**). The intensities of X-ray fluorescence from Ca (**G**–**I**) and P (**M**–**O**) on the arrow in (**A**–**C**) are displayed in (**G**–**I**) and (**M**–**O**). In 2 and 4 weeks after implantation, calcium and phosphorus deposits were observed on the phosphorylated pullulan surface and surrounding new bone (**E**,**F**,**K**,**L**). PPL: phosphorylated pullulan, TCP: β-TCP, *: trabecular bone Bar, (**A**–**F**,**J**–**L**): 50 μm (Morimoto et al. Frontiers in Bioengineering and Biotechnology, 2023, 11 [61]).

**Figure 7:**
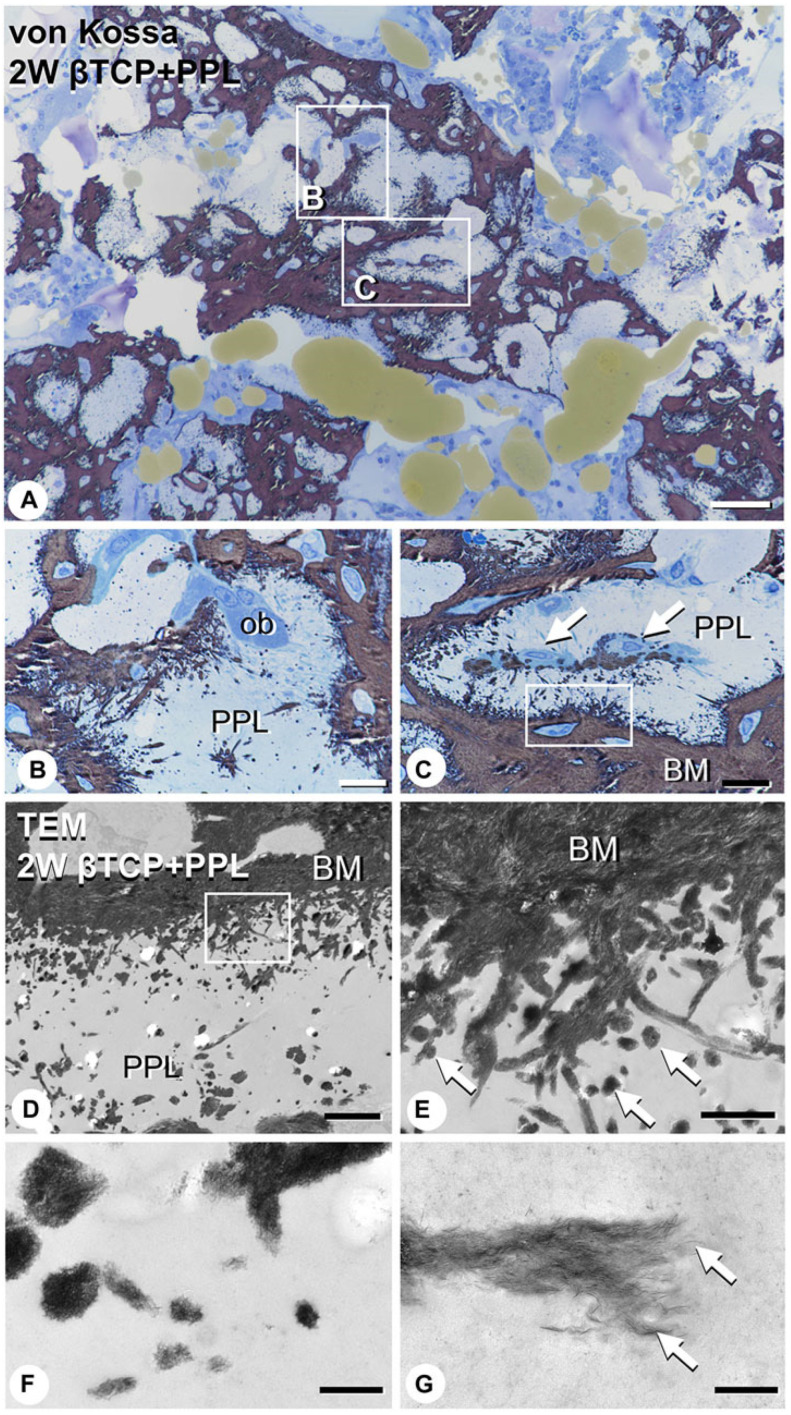
Von Kossa-stained images and transmission electron microscope images after the implantation of β-TCP and phosphorylated pullulan in rats with a tibia bone defect. (**A**): the low magnified image of von Cossa, (**B**,**C**): the high magnified image on the frame part in (**A**), arrow shows osteoblasts, (**D**): the low magnified TEM image, (**E**): the high magnified image on the frame part in (**D**), arrow shows calcified nodules, (**F**,**G**): the high magnified image from (**E**), arrow shows fine needle-like mineral crystals, PPL: phosphorylated pullulan, BM: bone matrix, ob: osteoblast Bar, (**A**): 50 μm, (**B**,**C**): 10 μm, (**D**): 3 μm, (**E**): 1 μm, (**F**): 0.3 μm, (**G**): 0.2 μm. (Morimoto et al. Frontiers in Bioengineering and Biotechnology, 2023, 11 [61]).

In addition, Morimoto et al. evaluated calcification related to phosphorylated pullulan using the elemental mapping of calcium and phosphorus using an electron probe micro-analyzer. Calcium and phosphorus were not detected in the phosphorylated pullulan 1 week after implantation, but 2 to 4 weeks after implantation, calcium and phosphorus deposits were observed on the phosphorylated pullulan surface and the surrounding new bone. Although the X-ray fluorescence intensity of phosphorus on phosphorylated pullulan 2 and 4 weeks after implantation did not differ, the X-ray fluorescence intensity of calcium increased over time (Figure 6). These results suggested that calcium might accumulate in phosphorylated pullulan and the surrounding new bone over time. Microstructural analysis revealed the formation of numerous calcite spheres and needle-like calcified crystal masses on the surface of pullulan phosphate (Figure 7), suggesting that phosphorylated pullulan may have an affinity for crystalline calcium and may promote calcification.

Based on these results, Morimoto et al. speculated that phosphorylated pullulan mixed with β-TCP exhibited osteoblast anchorage and osteoconductive ability as a scaffold material and might induce calcification by retaining calcium.

## 9. Application of Phosphorylated Pullulan to Areas Other than Hard Tissue

Phosphorylated pullulan is promoted for practical use in fields other than hard tissue. The submucosal injection material used for the endoscopic resection of gastric and other cancers is one of them. Satomi et al. confirmed that submucosal injection material-based phosphorylated pullulan could demonstrate a tissue elevation volume and duration comparable to existing submucosal injection materials using pigs and has high operability [62]. From these characteristics, the submucosal injection material containing phosphorylated pullulan as a key component has been approved under the Pharmaceutical Affairs Law in Japan and is marketed as “enRise”.

## 10. Conclusions

Phosphorylated pullulan is an unprecedented bioabsorbable material with bone adhesion properties developed based on experience in basic research on dental adhesive materials. It also can assist with bone formation. From these characteristics, phosphorylated pullulan is expected to be a novel bone replacement material. Currently, clinical trials of this material will begin in 2024 as a Class IV medical device, which is defined by the Japanese Act on Securing Quality, Efficacy, and Safety of Products Including Pharmaceuticals and Medical Devices. In addition to bone, phosphorylated pullulan is also expected to be used as a scaffold for the regeneration of various organs, as a carrier for drug delivery systems, and in a wide range of applications as a bioabsorbable material able to replace collagen and hyaluronic acid.

## Figures and Tables

**Table 1:** Comparison of bioabsorbable polymers.

	Natural Bioabsorbable Polymer		Synthetic Bioabsorbable Polymer		New Bioabsorbable Polymer
	Collagen	Hyaluronic Acid	Polyglycolic Acid	Polylactic Acid	Phosphorylated Pullulan
Biocompatibility	Good	Good	Cause inflammation during degradation	Cause inflammation during degradation	Good
Adhesion	Poor	Adhesion to wet tissue	Poor	Poor	Good
Gamma sterilization	Poor	Poor	Poor	Poor	Good
Manufacturing Method	Animal-derived	Animal-derived	Synthesis	Synthesis	Non-animal-derived

**Table 2:** Application development of phosphorylated pullulan.

Clinical Application	Study	Materials and Methods	Note
Dental implant	Cardoso et al. (2014) [53]	Titanium plates treated with phosphorylated pullulan were implanted in the rabbit tibia.	The bone fraction in areas 100 μm remote from the implant surface was higher than in the water treatment.
	Cardoso et al. (2017) [54]	Phosphorylated pullulan-coated implants were implanted in the pig skull bone.	The titanium implant surface with phosphorylated pullulan could improve the mineralization of the implant–bone interface.
	Cardoso et al. (2017) [55]	Titanium implant treated with phosphorylated pullulan were implanted in the pig parietal bone.	The peri-implant bone formation and bone-to-implant contact were improved, compared to the water-treated group.
	Nagamoto et al. (2024) [56]	Cell culture on a titanium disk coated with phosphorylated pullulan.	Cell proliferation and calcification were improved by coating with phosphorylated pullulan.
Pulp-capping material	Pedano et al. (2018) [57]	Preparing the hydraulic calcium-silicate cement including phosphorylated pullulan and culturing human dental pulp cells using eluates from this cement.	The eluate of this cement stimulated the proliferation, migration, and odontogenic differentiation of human dental pulp cells.
	Pedano et al. (2020) [58]	An injectable phosphopullulan-based calcium-silicate cement was used for the pulp-capping material ex vivo and in vivo.	This cement stimulated the formation of fibrous tissue and mineralized foci ex vivo and promoted the inflammatory reaction and regeneration of the pulp–tissue interface.
	Islam et al. (2024) [59]	Calcium hydroxide including phosphorylate pullulan was applied to rat first molar cavities.	Calcium hydroxide including phosphorylate pullulan could have the potential to minimize pulpal inflammation and to promote mineralized tissue formation.
Bone replacement material	Takahata et al. (2015) [60]	A mixture of phosphorylated pullulan and β-TCP was implanted in the medullary cavity of a mouse femur, a rabbit ulnar, and porcine vertebral body bone.	Phosphorylated pullulan and β-TCP promoted bone remodeling when phosphorylated pullulan and β-TCP were used together.
	Morimoto et al. (2023) [61]	A mixture of phosphorylated pullulan and β-TCP was implanted in rats with a tibia bone defect.	Phosphorylated pullulan mixed with β-TCP exhibited osteoblast anchorage and osteoconductive ability as a scaffold material and might induce calcification by retaining calcium

## Data Availability

The raw data supporting the conclusions of this article will be made available by the authors on request.
